# Sensor Modeling and Calibration Method Based on Extinction Ratio Error for Camera-Based Polarization Navigation Sensor

**DOI:** 10.3390/s20133779

**Published:** 2020-07-06

**Authors:** Haonan Ren, Jian Yang, Xin Liu, Panpan Huang, Lei Guo

**Affiliations:** 1 School of Automation Science and Electrical Engineering, Beihang University, Beijing 100191, China; renhaonan@buaa.edu.cn (H.R.); xliubuaa@buaa.edu.cn (X.L.); lguo@buaa.edu.cn (L.G.); 2 Beijing Advanced Innovation Center for Big Data-Based Precision Medicine, School of Medicine and Engineering, Beihang University, Beijing 100191, China; 3 Key Laboratory of Big Data-Based Precision Medicine (Beihang University), Ministry of Industry and Information Technology, Beijing 100804, China; 4 Hangzhou Innovation Institute, Beihang University, Hangzhou 310051, China; z5042207@zmail.unsw.edu.au

**Keywords:** polarization sensor, inconsistency of the extinction ratio, sensor model, calibration model, degree of polarization error

## Abstract

The performance of camera-based polarization sensors largely depends on the estimated model parameters obtained through calibration. Limited by manufacturing processes, the low extinction ratio and inconsistency of the polarizer can reduce the measurement accuracy of the sensor. To account for the challenges, one extinction ratio coefficient was introduced into the calibration model to unify the light intensity of two orthogonal channels. Since the introduced extinction ratio coefficient is associated with degree of polarization (DOP), a new calibration method considering both azimuth of polarization (AOP) error and DOP error for the bionic camera-based polarization sensor was proposed to improve the accuracy of the calibration model parameter estimation. To evaluate the performance of the proposed camera-based polarization calibration model using the new calibration method, both indoor and outdoor calibration experiments were carried out. It was found that the new calibration method for the proposed calibration model could achieve desirable performance in terms of stability and robustness of the calculated AOP and DOP values.

## 1. Introduction

There have been many studies showing that insects such as desert ants and butterflies are able to determine their heading-orientation based on the skylight polarization patterns [1,2,3,4,5]. Inspired by the polarization navigation strategy of these insects, the bioinspired polarization navigation methods have attracted much attention in the field of autonomous navigation. Unlike traditional navigation methods—Including the Inertial Navigation System (INS) and Global Navigation Satellite System (GNSS)—The bioinspired polarization navigation method does not suffer from accumulation errors and electromagnetic interference [6,7]. It has been widely studied in the fields of robotics, UAVs and other unmanned systems [8,9].

Inspired by the polarization vision mechanism of insects, a variety of polarization sensors have been developed. In general, the polarization sensors can be divided into two types: polarization-sensitive type (POL-type) sensor and camera-based polarization sensor. The POL-type sensor simulates the signal process in the polarization opponent neurons in ommatidium of insects [10,11,12,13]. Although the POL-type sensor is able to generate real-time navigation measurements with less computational complexity, it can be severely affected by the cloudy sky, the tree occlusion, and other complex outdoor sky conditions [14,15,16]. In contrast, the camera-based polarization sensor can measure multiple direction information of polarization pattern and achieve satisfactory accuracy in partly sky conditions [17,18]. In the last decades, there have been many camera-based polarization sensors developed for different applications. For instance, Mukul et al. presented a polarization navigation method using the Stokes parameters to determine the orientation. The sensor used a metallic wire grid micro-polarizer to acquire position information [19]. Powel et al. developed a fully polarized sensing array to obtain the all-sky atmospheric polarization pattern. A calibration method based on the polarimeter behavior was established. To account for the errors caused by the fabrication of the imaging array nanowires, one corresponding calibration method was proposed. This method has been demonstrated to be able to significantly reduce the root mean square error (RMSE) of degree of polarization (DOP) with each super-pixel calibrated as a unit [20,21]. Wolfgang et al. presented a lightweight polarization sensor consisting of four synchronized cameras, which could determine the solar elevation angle even in the presence of clouds for UAVs [22,23]. Wang et al. designed a real-time bionic camera-based polarization navigation sensor with two working modes including one of single-point measurement mode and the other one of multi-point measurement mode. The azimuth of polarization (AOP) of the sensor can reach up to 0.3256° accuracy [24,25]. Sun et al. proposed three improved models of imaging and analyzed different polarization pattern distortion degrees of these imaging models [26]. Zhao et al. proposed a polarization imaging algorithm containing a pattern recognition algorithm and one orientation measurement algorithm to reduce the computational load. The proposed imaging algorithm was verified with simulations and experiments for utilization in embedded systems with low computational load [27]. Hu et al. designed and tested a bionic camera-based polarization navigation sensor. The standard deviation of the AOP calibrated indoors is 0.0516° [28]. In 2017, they designed a new microarray camera-based polarization compass which was calibrated based on iterative least squares method. The standard deviation of the AOP calibrated is 0.06° achieved under indoor conditions [29]. Aycock et al. designed an image-based sky AOP sensing system which could be used in GPS-challenged environments with high-precision navigation [30,31].

The aforementioned camera-based polarization sensors can all provide heading information using the AOP and DOP measurements without GNSS. However, the AOP and DOP calculations do not take into account the extinction ratio inconsistency of the polarizer, which could cause conformance degradation in the light intensity of two orthogonal channels, and result in decreasing accuracy and stability of the measured AOP and DOP. In addition, the existing calibration models for camera-based polarization sensors, only AOP error is considered to estimate the model parameters without considering the effect of DOP error on the accuracy of calibration model [24,28,29,32,33].

In this study—To account for the extinction ratio inconsistency and enhance the calibration performance for the camera-based polarization sensor—One extinction ratio coefficient was introduced into the polarization calibration model. Then, a calibration method considering both AOP and DOP errors is proposed to improve the accuracy of the calibration model. The main technical features can be summarized as follows:

(1) The influence of the inconsistency of the extinction ratio between orthogonal polarizers on sensor was quantitative analyzed. Accordingly, a new camera-based polarization sensor model based on the extinction ratio coefficient was established. As such, the light intensity of the two orthogonal channels could be unified with the aid of extinction ratio coefficient. Unlike the existing polarization sensor model [26,28,29], a model with extinction ratio parameter was considered as a fine structure model. Moreover, the effect of extinction ratio error on the sensor was carefully analyzed.
(2) A new calibration method integrated both the AOP and DOP error is proposed. With the addition of DOP error, the estimation accuracy of sensor calibration model parameters was further improved. Meanwhile the stability and robustness of AOP and DOP could be improved simultaneously.

The remainder of this study is organized as follows. In Section 2, the bionic camera-based polarization sensor structure and the corresponding AOP and DOP calculation are reviewed. In Section 3, the polarization calibration model with the extinction ratio coefficient and the calibration method based on both AOP and DOP error are described. To evaluate the calibration performance with the proposed calibration method, the results of both indoor and outdoor calibration experiments are analyzed in Section 4. Finally, concluding remarks are presented.

## 2. Bionic Camera-Based Polarization Sensor

### 2.1. Polarization Sensor Structure

**Figure 1:**
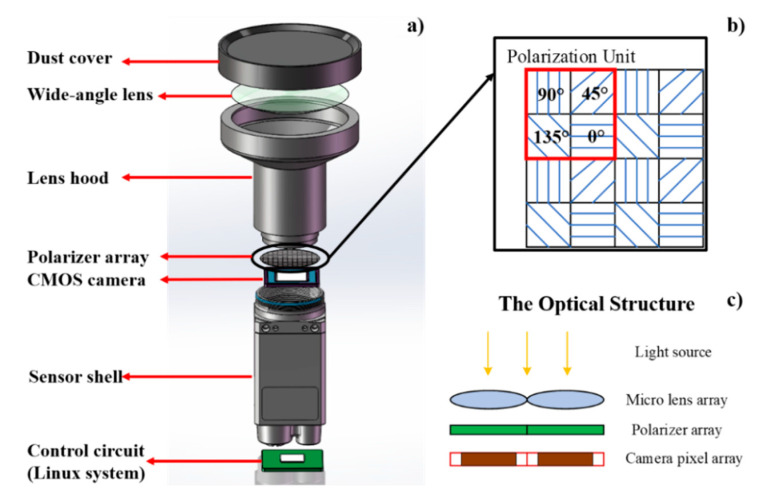
Bionic polarized-sensor structure diagram. (**a**) Bionic polarization sensor hardware component; (**b**) one example of the polarization unit with different installation angle; (**c**) the optical structure of the sensor.

In this study, the camera-based polarization sensor designed by Space Intelligent Autonomous Systems Research Center in Beihang University is used. It consists of one lens layer, one polarization optical information acquisition layer and one photosensitive circuit layer, as shown in Figure 1.

(1) The lens layer is used to sense the polarization pattern with a wide field of view. The FE185C057HA-1 wide-angle lens is adopted. Its focal length is 1.8 mm, and the field of view is about 185° × 185°.
(2) The polarization optical information acquisition layer is used to detect the optical characteristics of polarized skylight. A pixel-level polarization complementary metal oxide semiconductor (CMOS) camera (Sony IMX250MZR) is employed as shown in Figure 1a. The polarization camera includes a CMOS where 2 × 2 matrices of polarizers are used in front of every 2 × 2 photosensors. The array polarizer contains 2048 × 2448-pixel channels to measure the polarized skylight, and the size of each pixel channel is 3.45 × 3.45 μm^2^. As shown in Figure 1b, four adjacent pixel channels constitute a polarization unit, and the polarizer installation directions of corresponding channels are 0°, 45°, 90° and 135°, respectively. In addition, the CMOS camera is used to acquire the polarized skylight intensity information.
(3) The control and processing circuit layer ensures real-time acquisition and processing of polarized images obtained by the CMOS camera. It consists of a control module, a communication module and a memory module. In the control module, a Linux system is used to process the polarized skylight intensity images to finally obtain polarization information. Finally, the sun vector information is obtained according to the vertical relationship between the sun vector and the polarization vector, which is stored in the memory module.

### 2.2. Polarization Calculation

According to the Malus law, the light-intensity response value from the pixel channels in the same polarization unit can be expressed by [34]:

(1) $$Ip_{i}=0.5I\left[1+d\cos\left(2\varphi -2\alpha_{i}\right)\right]$$

where i=1,…,4 is i–th pixel channel of one polarization unit; Ip is the output light intensity through the polarizer; I is intensity of incident light; d is the DOP of the incident light; φ is the angle between the polarization direction of the incident light and the reference direction; and α is the angle between the polarizer installation direction and the reference direction of each pixel channel in a polarization unit.

According to the light-intensity response value derived from each pixel channel, the AOP and DOP can be calculated using the Stokes parameters, as follows [29,34]:

(2) $$\begin{array}{l} AOP=0.5\tan^{-1}\left(S_{2}/S_{1}\right)\\ DOP=\sqrt{S_{1}^{2}+S_{2}^{2}}/S_{0} \end{array}$$

where $S_{0}$, $S_{1}$ and $S_{2}$ are components of the incident light Stokes vector.

## 3. Camera-Based Polarization Sensor Calibration

The camera-based polarization sensor calibration includes the establishment of calibration model and the improvement of calibration method. First, the calibration model of the sensor is improved for extinction ratio and other error sources. Then, the calibration model coefficients are estimated by improving the existing calibration method.

### 3.1. Sensor Model of Camera-Based Polarization Sensor

As an important parameter of polarization sensor, extinction ratio directly affects the accuracy and stability of the obtained polarization information. In addition, the performance of the sensor is also limited by the structure error of polarizer, the photographic error of camera, the environmental error, etc. To account for these errors, a new calibration model, including the array polarizer model and the CMOS camera photosensitivity model, is established by using different error parameters.

#### 3.1.1. Array Polarizer Model with Extinction Ratio

As a key component of the camera-based polarization sensor, the polarizer is adopted to convert the nature light to the polarized light for heading determination. However, in the manufacturing process, the extinction ratio of microarray polarizer is limited by the influence of the fine grid design [35,36,37]. The low extinction ratio can affect the accuracy of the polarization sensor. In our sensor used, the extinction ratio of the array polarizer is about 24 dB (250:1 approximately). Therefore, the measurement model of polarization sensor needs to be calibrated with high precision according to extinction ratio [37]. Moreover, we proposed the sensor calibration model with extinction ratio to adapt to the variation of measurement information caused by extinction ratio attenuation and inconsistency.

The effect of extinction ratio is analyzed using a single pixel channel. According to the literature [34,38,39,40,41], the extinction ratio of polarizer can be expressed as follows:

(3) $$ER=10\log_{10}\left(I_{\max}/I_{\min}\right)$$

where ER represents extinction ratio of the polarization sensor, which is a constant [35,37]; and $I_{\max}$ and $I_{\min}$ are the maximum and minimum values of the sine curve of the light-intensity response, respectively.

According to Equation (1), $I_{\max}$ and $I_{\min}$ can also be expressed as:

(4) $$I_{\max}=I\left(1+\tilde{d}\right)\;,\;I_{\min}=I\left(1-\tilde{d}\right)$$

According to Equations (3) and (4), the relationship between ER of the polarizer and DOP of the incident light can be obtained:

(5) $$\tilde{d}=(10^{ER/10}-1)/(10^{ER/10}+1)$$

In order to ensure that the calculated DOP value is the same for every polarization unit with the same incident light, the extinction ratio coefficient σ can be introduced:

(6) $$\tilde{d}=\sigma d$$

The polarization sensor calibration model can then be changed to:

(7) $$Ip=0.5I\left(1+\tilde{d}\cos\left(2\varphi -2\alpha \right)\right)=0.5I\left(1+\sigma d\cos\left(2\varphi -2\alpha \right)\right)$$

**Figure 2:**
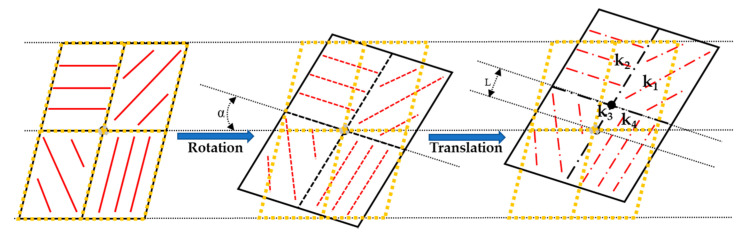
The polarizer installation misalignment of one polarization unit.

Besides the ER inconsistency of polarizer, the installation misalignment between the array polarizer and the CMOS camera photosensor also affects the accuracy of obtained polarization information [28,29]. It mainly includes the rotation and translation installation misalignments, which could result in optical path information coupling, as shown in Figure 2. The black and yellow 2 × 2 matrices represent the array polarizers and photosensors of CMOS camera, respectively.

Due to the rotation and translation misalignments, the light intensity acquired by every pixel channel of CMOS camera is no longer from a single channel, but the coupling light intensity from the four channels. Suppose that the intensity of the four pixel channels of one polarization unit are $Ip_{1}$, $Ip_{2}$, $Ip_{3}$ and $Ip_{4}$, respectively. The corresponding intensity distribution coefficients of the four pixel channels are $k_{1}$, $k_{2}$, $k_{3}$ and $k_{4}$. Then, the output light intensity of the polarization response perceived by every pixel channel is:

(8) $$\begin{array}{c} Ip=k_{1}Ip_{1}+k_{2}Ip_{2}+k_{3}Ip_{3}+k_{4}Ip_{4}\\ =0.5I\left[1+\eta d\cos\left(2\varphi -2\left(\alpha +\Delta \right)\right)\right] \end{array}$$

where $\sum_{j=1}^{4}k_{j}=1$, α is the ideal installation angle direction of the polarizer, with $\alpha_{1}$, $\alpha_{2}$, $\alpha_{3}$ and $\alpha_{4}$ respectively set to 0°, 45°, 90° and 135°; and Δ is installation angle error of every pixel channel of polarization unit; and η is the parameter introduced to account for the optical path coupling and ER inconsistency, which can be calculated as η=ξ×σ with ξ representing the optical path coupling coefficient, and σ the extinction ratio coefficient.

Furthermore, the attenuation effects of different polarization channel on incident polarized light are considered in the array polarizer model, which can be expressed as:

(9) $$Ip_{i}=\beta_{i}I\left[1+\eta_{i}d\cos\left(2\varphi -2{\tilde{\alpha }}_{i}\right)\right]+v_{i}$$

where $\beta_{i}$ represents the attenuation parameter of incident light intensity of the i–th pixel channel; and ${\tilde{\alpha }}_{i}$ represents installation angle with error, which can be calculated as ${\tilde{\alpha }}_{i}=\alpha_{i}+\Delta_{i}$; and $v_{i}$ denotes the Gaussian noise of measurement.

#### 3.1.2. CMOS Camera Photosensitivity Model

A CMOS camera below the array polarizer was used to collect light-intensity information. Due to difference of photodiodes and thermal effect of circuit, the photosensitivity of every pixel channel on the CMOS camera is inconsistent, which could lead to decrease on measurement accuracy of polarization sensor [28,29]. To account for the photosensitivity error, the mapping relationship between incident light intensity and the CMOS camera response light intensity can be modeled as:

(10) $$I{\mathrm{cmos}}_{i}=\lambda_{i}Ip_{i}+\delta_{i}$$

where i is the i–th pixel channel of CMOS camera; $\lambda_{i}$ is the proportionality coefficient; $\delta_{i}$ is the bias coefficient; $I{\mathrm{cmos}}_{i}$ is the light-intensity response value of i–th pixel channel; and $Ip_{i}$ is the output light-intensity value after passing through the i–th polarizer. This model is referred to as the CMOS camera photosensitivity model in this study.

### 3.2. Calibration Method

The parameters of the bionic polarization sensor calibration models, including the CMOS camera photosensitivity model parameters λ,δ and the array polarizer model parameters $\beta ,\eta ,\tilde{\alpha }$, are calibrated in two steps. The first step (Step 1) is to calibrate the CMOS camera photosensitivity model, and the second step (Step 2) is to calibrate the array polarizer model. Both AOP and DOP errors are taken into account during the following calibration method.

#### 3.2.1. The CMOS Camera Photosensitivity Model Calibration

Using the uniform unpolarized light source of the integrating sphere, light-intensity response images under different incident light intensity are collected by the CMOS camera. According to Equation (10), the light-intensity responses of all pixel channels of the CMOS camera can be expressed as:

(11) $$Y=HX=\left[\begin{array}{cc} Ip_{1} & 1\\ \vdots & \vdots \\ Ip_{k} & 1 \end{array}\right]\left[\begin{array}{c} \lambda_{i}\\ \delta_{i} \end{array}\right]$$

where $Y={\left[\begin{array}{ccc} I{\mathrm{cmos}}_{1}^{i} & \cdots & I{\mathrm{cmos}}_{k}^{i} \end{array}\right]}^{T}$ is CMOS camera light-intensity response matrix; $X={\left[\begin{array}{cc} \lambda_{i} & \delta_{i} \end{array}\right]}^{T}$ denotes calibration coefficient vector; and $H=\left[\begin{array}{cc} Ip_{1} & 1\\ \vdots & \vdots \\ Ip_{k} & 1 \end{array}\right]$ is the incident light-intensity matrix; and k denotes the number of the incident light intensity.

The coefficients of the CMOS camera photosensitivity model can be estimated based on the linear least square estimation algorithm as:

(12) $$X={\left(H^{T}H\right)}^{-1}H^{T}Y$$

#### 3.2.2. The Array Polarizer Model Calibration

To calibrate the array polarizer model, the bionic polarization sensor is fixed on a high-precision rotary platform. Polarization light source from the integrated ball is used. The reference direction is defined as the installation direction of polarizer 0° channel under the initial state of the turntable. The rotation angle of the rotary platform relative to the reference direction is used as a benchmark to calibrate the array polarizer model. For every polarization unit of polarizers, the polarization response can be written as:

(13) $$\vec{I}=H\vec{x}=\left[\begin{array}{ccc} \beta_{1} & \eta_{1}\beta_{1}\cos2{\tilde{\alpha }}_{1} & \eta_{1}\beta_{1}\sin2{\tilde{\alpha }}_{1}\\ \beta_{2} & \eta_{2}\beta_{2}\cos2{\tilde{\alpha }}_{2} & \eta_{2}\beta_{2}\sin2{\tilde{\alpha }}_{2}\\ \beta_{3} & \eta_{3}\beta_{3}\cos2{\tilde{\alpha }}_{3} & \eta_{3}\beta_{3}\sin2{\tilde{\alpha }}_{3}\\ \beta_{4} & \eta_{4}\beta_{4}\cos2{\tilde{\alpha }}_{4} & \eta_{4}\beta_{4}\sin2{\tilde{\alpha }}_{4} \end{array}\right]\left[\begin{array}{c} I\\ Id\cos2\varphi \\ Id\sin2\varphi \end{array}\right]$$

where $\vec{I}={\left[\begin{array}{cccc} Ip_{1} & Ip_{2} & Ip_{3} & Ip_{4} \end{array}\right]}^{T}$ is the light-intensity vector of the polarization response; $\vec{x}$ is the vector about polarization of the incident light; and H is the calibration coefficient matrix of the array polarizer model.

Assuming that m groups of polarization response images are obtained, the polarization information vector group X and intensity vector group $Q_{n}$ of n–th polarization unit are expressed as:

$$X={\left[\begin{array}{ccc} {\vec{x}}_{1} & \ldots & {\vec{x}}_{m} \end{array}\right]}_{3\times m},\;Q_{n}={\left[\begin{array}{ccc} {\vec{I}}_{1} & \ldots & {\vec{I}}_{m} \end{array}\right]}_{4\times m}$$

The initial calibration coefficient matrix $H_{n}$ of the array polarizer model is estimated based on the linear least square algorithm:

(14) $$H_{n}^{T}={\left(XX^{T}\right)}^{-1}XQ_{n}{}^{T}$$

The initial calibration coefficient of polarizer model can then be calculated with the components of $H_{n}$ as follows:

(15) $$\begin{array}{l} \beta_{i}^{n}=H_{n}\left(i,1\right)\\ \alpha_{i}^{n}=0.5\tan^{-1}\left(H_{n}\left(i,3\right)/H_{n}\left(i,2\right)\right)\\ \eta_{i}^{n}=\sqrt{{\left(H_{n}\left(i,2\right)\right)}^{2}+{\left(H_{n}\left(i,3\right)\right)}^{2}}/H_{n}\left(i,1\right) \end{array}$$

Inspired by the calibration method used for the POL-type polarization sensor considering both AOP and DOP errors [8], a least-squares iterative calibration algorithm also taking into account these two errors is proposed for the bionic camera-based polarization sensor, to account for the extinction ratio inconsistency that is associated with measurement accuracy of DOP. Compared with the existing calibration method based on AOP [6,11,28,29], the innovation of this calibration method is to establish the relationship between extinction ratio and DOP error.

The calibration parameters vector is defined as:

(16) $$x={\left[\begin{array}{cccccccccccc} \beta_{1} & \beta_{2} & \beta_{3} & \beta_{4} & \eta_{1} & \eta_{2} & \eta_{3} & \eta_{4} & \alpha_{1} & \alpha_{2} & \alpha_{3} & \alpha_{4} \end{array}\right]}^{T}$$

The AOP and DOP errors can be expressed as:

(17) $$f\left(x\right)=\left[\begin{array}{c} \varphi_{n}-\varphi \left(x\right)\\ d_{n}-d\left(x\right) \end{array}\right]$$

where $\varphi_{n}$ and $d_{n}$ are the reference values of the AOP and DOP, respectively; φ(x) and d(x) are the corresponding estimated values calculated based on Equation (2).

An objective function in the form of the squared Euclidean norm of the error f(x) is established as follows:

(18) $$x=\arg\underset{x}{\min}\left({\left\|f\left(x\right)\right\|}_{2}^{2}\right)$$

To solve this objective function, the secant Levenberg–Marquardt (LM) least square algorithm is used to obtain the optimized estimation [42,43]. The initial value of the calibration parameters can be obtained based on Equation (15).

## 4. Experiment Results and Analysis

In this section, one simulation experiment was carried out to first analyze the influence of the extinction ratio on calibration model. In addition, both indoor and outdoor calibration experiments were investigated to validate the effectiveness of the proposed calibration model and calibration method.

### 4.1. Simulation Experiment for Extinction Ratio Analysis

**Figure 3:**
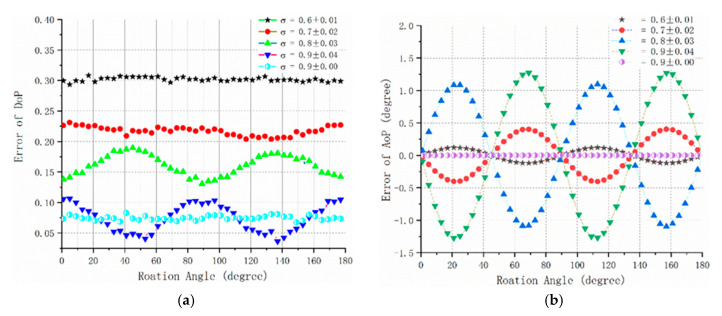
Effect of extinction ratio coefficient on the sensor simulation diagram. (**a**) degree of polarization (DOP) error; (**b**) azimuth of polarization (AOP) error.

The extinction ratio coefficient used in the simulation experiment was set to be 0.6, 0.7, 0.8 and 0.9, respectively. In theory, the greater the extinction ratio coefficient, the better the extinction characteristics of the polarizer (When the extinction ratio coefficient is 1, the extinction ratio of the polarizer is ideal value 1/∞). To better illustrate the effect of the ER inconsistency of the pixel channel on the compass precision, random errors were added to the different extinction ratio coefficients. The magnitudes of the added random errors were ±0.01, ±0.02, ±0.03, ±0.04 and ±0.00, respectively. The simulation results are shown in Figure 3.

As can be seen from the simulation results, the accuracy of DOP is positively correlated with extinction ratio of polarizer. When the extinction ratio coefficient decreases from 0.9 to 0.8, the mean error of DOP increases from 7.5% to 15%. Moreover, the inconsistency of ER can cause the fluctuation of polarization error. The standard deviation of both DOP error and AOP error is enlarged almost three times with the increase in two orders of magnitude of the extinction ratio coefficient error. Hence, extinction ratio as an important parameter, must be considered in the process of sensor calibration.

### 4.2. Indoor Calibration Experiment

#### 4.2.1. Calibration Results

**Figure 4:**
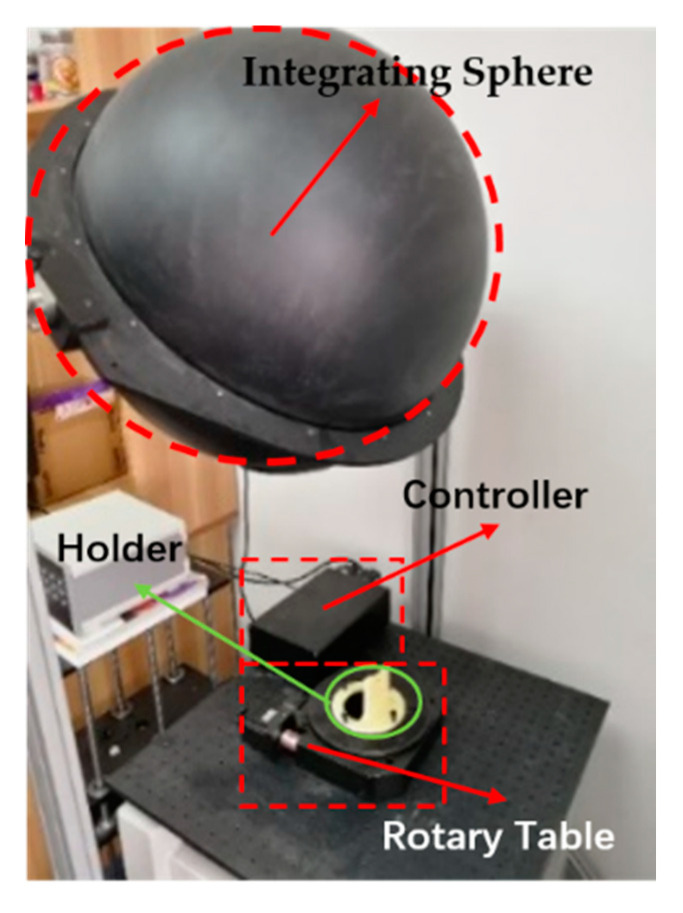
Indoor calibration experiment environment.

The setup used for the indoor calibration experiment include a high-precision electric rotary platform, an integrating sphere, an optical platform and a polarizer, as shown in Figure 4. The integrating sphere with high extinction ratio polarizer can provide high quality linear polarization light source for the sensor. The bionic polarization sensor was installed on an electric rotary platform that was fixed on an optical platform. The rotary platform was used to provide polarized light source with direction change for the bionic polarization sensor with the 0.01° rotating accuracy.

**Step 1**: Calibration experiment of CMOS camera photosensitivity model. In order to reduce the interference of stray light, the bionic polarization sensor was placed in a closed environment with only the integrating sphere light source. The photosensitivity model of camera was calibrated with natural light source. The exposure time of the camera was set to a constant value. By changing the light intensity of the output natural light from the integrating sphere, the camera was controlled to collect response images at 30 lux intervals within the range of 20–680 lux. Moreover, a total of 23 sets of experimental data were obtained.

**Step 2:** Calibration experiment of the array polarizer model. The same experiment environment as the CMOS camera calibration experiment was used for the calibration experiment of the array polarizer. By rotating the rotary platform at 3° intervals from 0° to 360°, 120 sets of experimental data were collected. The rotation angle of the platform was used as the external direction reference for the array polarizer model calibration of the bionic polarization sensor. By changing the intensity of the output polarized light from the integrating sphere, 120 groups of experimental data were collected under the radiation intensity of 700lux, 500lux, 350lux and 140lux, respectively.

**Figure 5:**
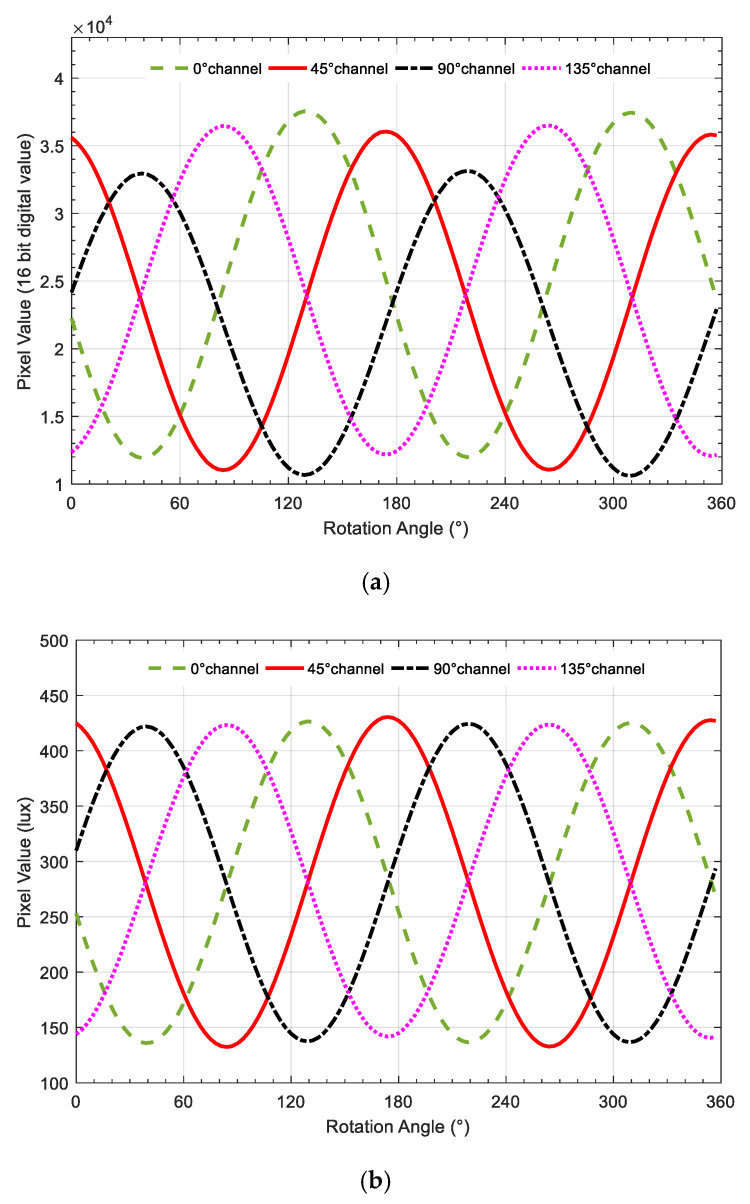
Indoor calibration experiment diagram. (**a**) four-channel light-intensity response curve before calibration; (**b**) four-channel light-intensity response curve before calibration.

The method described in equation (12) is used to calibrate the CMOS camera photosensitivity model. The average light-intensity value of CMOS camera under polarized light varies with the rotation angle of the rotary platform, as shown in Figure 5. Figure 5a,b illustrates the intensity responses before and after calibration, respectively. It can be found that the amplitude and bias of every pixel channel response curve are significantly different before calibration, which are of almost the same magnitude after calibration of the CMOS camera photosensitivity model.

**Figure 6:**
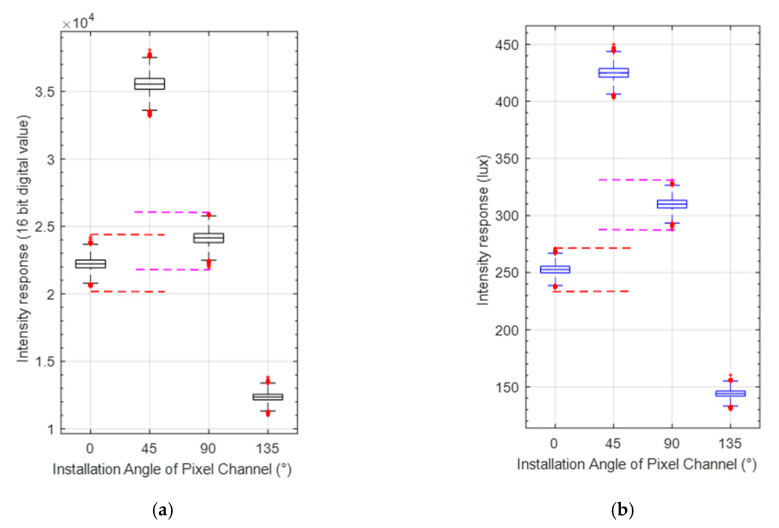
Distribution of light-intensity response of every pixel in single image (**a**) before calibration and (**b**) after calibration.

To further demonstrate the contribution of the calibration, Figure 6 gives the comparison of the distribution of light-intensity response of every pixel. As can be seen from Figure 6a,b, the response value of two pixel-channels with similar light-intensity response are more clearly distinguished after calibration. Due to the inconsistency of photosensitivity of CMOS camera, there is an overlap between the measurement value derived from the pixel channels of 0° and 90° as shown in Figure 6a, i.e., the crossed area formed by the red and magenta dotted lines. Moreover, after the photosensitivity inconsistency calibration, the two pixel channels have completely different intensity responses as shown in Figure 6b after calibration, i.e., the red and magenta dotted areas no longer cross, which will facilitate the calibration of extinction ratio error and installation error of polarizer.

**Figure 7:**
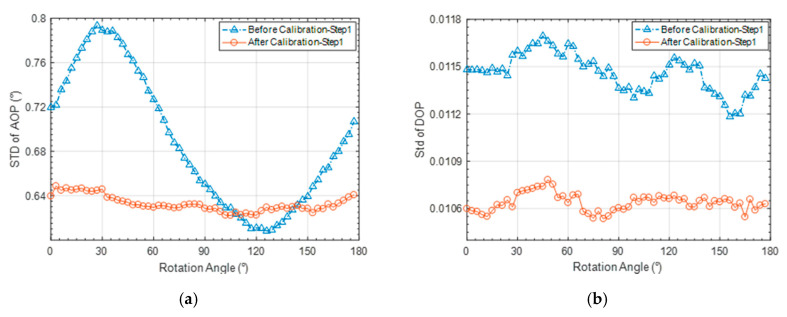
(**a**) AOP and (**b**) DOP SD comparison before and after calibration experiment of CMOS camera photosensitivity model.

The calibrated coefficients of the CMOS camera photosensitivity model were used to correct the original light-intensity data and the standard deviations (SD) of AOP and DOP of every image rotating with the rotary platform before and after calibration were compared, as shown in Figure 7. The standard deviations of AOP and DOP of every polarization unit are decreased by 8% and 7%, respectively; As can be seen from Figure 7a, the sinusoidal fluctuation of standard deviation of AOP in each image is greatly reduced after calibration. The standard deviation of AOP calculated within the rotation angle range of 110 to 130 before calibration is smaller than after calibration, which can be attributed to the positive and negative offsets when calculating AOP based on Equation (2).

**Figure 8:**
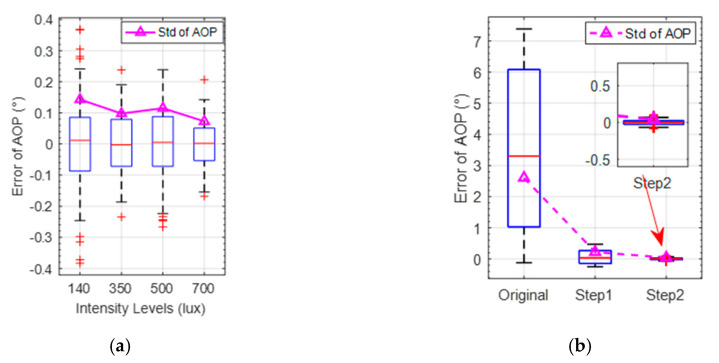
(**a**) AOP accuracy after calibration under different polarized light-intensity levels; (**b**) Comparison of SD of AOP error before and after calibration.

To demonstrate the influence of the incident light intensity on the calibration performance, the experiment was carried out under the incident polarized light with the different intensity levels of 700 lux, 500 lux, 350 lux and 140 lux. The CMOS camera was not overexposed under the maximum incident light intensity. As the exposure time and the rotation of the turntable could cause fluctuations of the photosensitive values derived from the pixel channels of every polarization unit, it is necessary to eliminate the influence of these factors during the calibration. A median filter was first applied to the light-intensity information of every pixel channel. The statistics of the calibrated AOP error values under different light-intensity levels of the same polarization unit are shown in Figure 8a and Table 1. It can be seen that the SD and the maximum absolute error (MAXE) tended to decrease as the intensity level of incident light increased.

**Figure 9:**
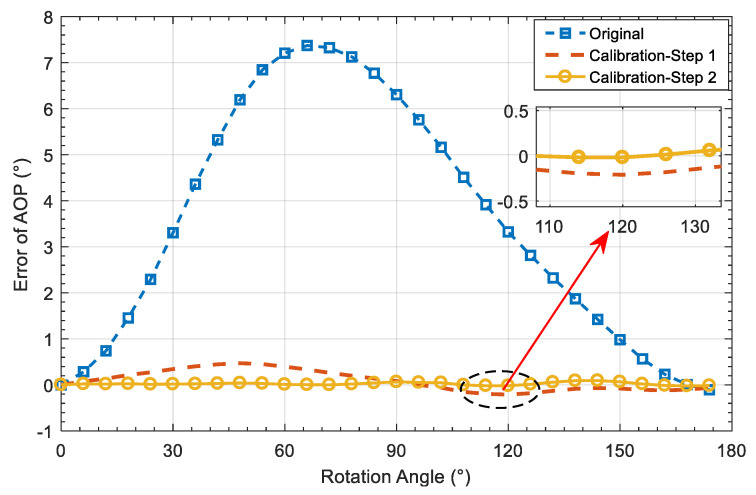
Comparison of AOP error before and after calibration.

The rotation angle of the platform is used as the orientation reference of the bionic polarization sensor. The mean AOP error calculated over all polarization units was used as the orientation index to evaluate the calibration performance of the sensor, which is shown in Figure 8b and Figure 9. It can be seen that the SD of original AOP is 2.62°. The SD is 0.22° after calibration of CMOS camera photosensitivity model, which is reduced by 91.6% compared with that before calibration. The SD is further reduced to 0.04° after the calibration of polarizer model, which is reduced by 81.8% compared to step1 calibration. In addition, the mean absolute error (MAE) is reduced to 0.03° after the Step 2 calibration. The detailed comparison of orientation error with and without calibration is shown in Table 2.

#### 4.2.2. Performance Analysis of Calibration Model and Method

To validate the effectiveness of the proposed calibration model with ER and calibration method base on both AOP error and DOP error, three different comparative experiments were designed, which are referred to as Case1, Case2 and Case3 in the following experiments, which are described in detail in Table 3.

**Figure 10:**
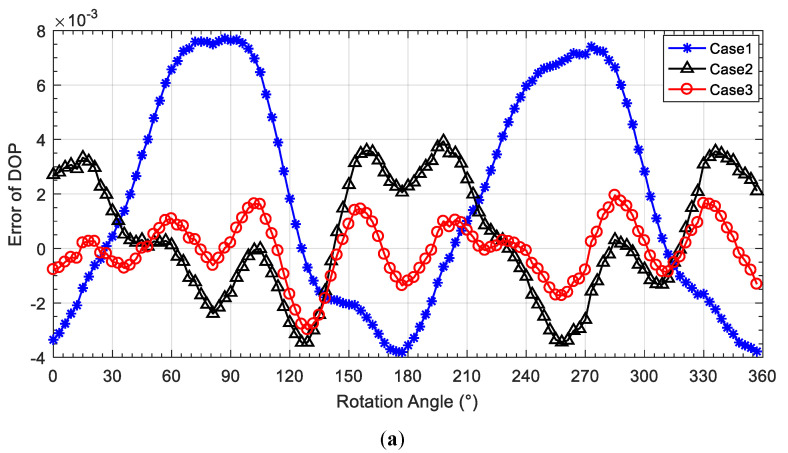

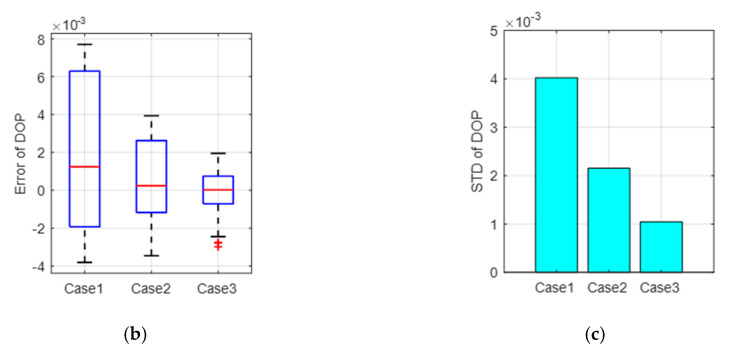
DOP-error comparison of three cases. (**a**) DOP-error curve comparison; (**b**) DOP error distribution; (**c**) SD of DOP-error comparison.

The calibration error curves of DOP of the three cases are shown in Figure 10a. Figure 10b,c shows the error distribution and SD comparison of DOP error of the three cases. It can be seen that the SD DOP error for Case3 is decreased by 74.1% compared with that for Case1 and 51.6% compared with that for Case2. The mean absolute error (MAE) of DOP for Case3 is decreased by 77.9% compared with that for Case1 and 55.4% compared with that for Case2. The detailed experimental results can be found in Table 4. It can be seen that the calibration method considering both AOP and DOP errors for the proposed calibration model with the ER coefficient can effectively improve the DOP calculation of the bionic polarization sensor.

**Figure 11:**
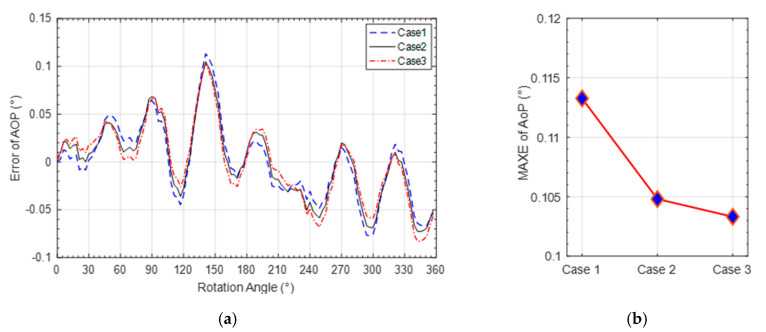
AOP-error comparison of three cases. (**a**) AOP-error curve comparison; (**b**) MAXE of AOP-error comparison.

The performance comparison of AOP calibration of the three cases is also shown in Figure 11a. Figure 11b shows the comparison of the maximum absolute error (MAXE) of AOP obtained by the three cases. Detailed AOP calibration accuracies are shown in Table 5. It can be seen that the maximum absolute error of AOP derived from Case3 is decreased by 8.8% compared with that of Case1 and 1.9% compared with that of Case2. By comparing the three cases, it can be seen that the MAE and the SD of AOP are close. In a word, the proposed calibration model and calibration method can improve the sensor performance, mainly improving the DOP measurement performance, which is beneficial to DOP based navigation.

### 4.3. Outdoor Calibration Experiment

**Figure 12:**
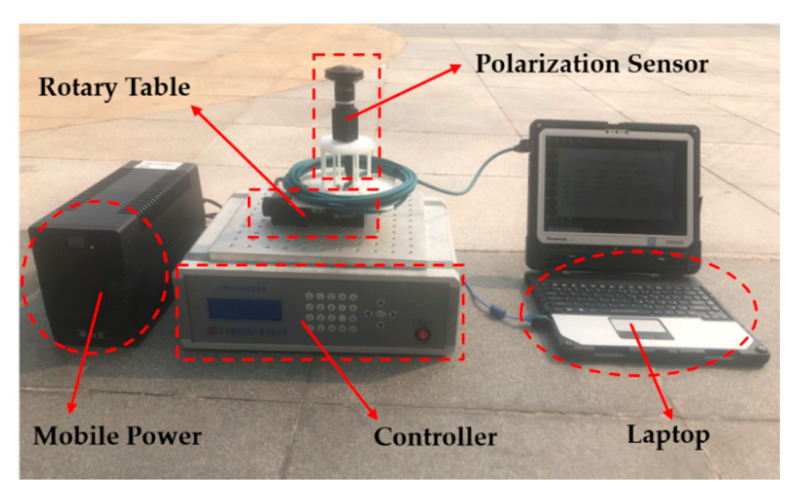
Setup for outdoor test.

To further validate the effectiveness of the proposed calibration model and calibration method, the bionic camera-based polarization sensor was calibrated and tested outdoors at 16:00 on January 3, 2020 and 8:00 on January 4, 2020. The experiment was conducted on the campus of Beihang University (longitude 116.3398°, latitude 39.9794°, altitude 63.90 m). The same setup was used for the outdoor test as shown in Figure 12. The polarization sensor was fixed on a horizontal electric platform, and a calibration software was used to control the electric platform to rotate from 0° to 360° at 3° intervals. The skylight images were collected and saved with every rotation.

A polarization unit was selected to analyze the accuracy of outdoor calibration navigation. The difference value between the rotation angle of the turntable and the variation of the azimuth of the sun is used as the benchmark of the AOP. The orientation error was defined as the difference between the AOP of the output of the polarization sensor and the benchmark.

**Figure 13:**
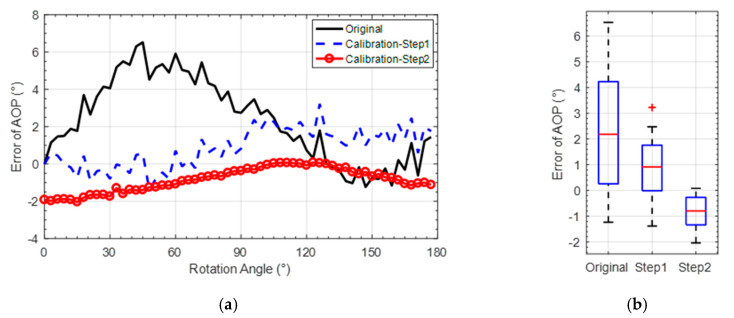
AOP-error comparison before and after calibration. (**a**) AOP-error curve comparison; (**b**) AOP error distribution.

Figure 13a,b show the performance comparison of AOP error before and after calibration in terms of variation and SD values. The detailed calibrated AOP results are shown in Table 6. Compared with the results obtained before calibration, the MAE of AOP is decreased by 51.6% and the SD decreased to 38.8% after the Step 1 calibration. Compared with the Step 1 calibration, the MAE of AOP is decreased by 48.5% and the SD decreased to 38.8% after calibration of the Step 2.

**Figure 14:**
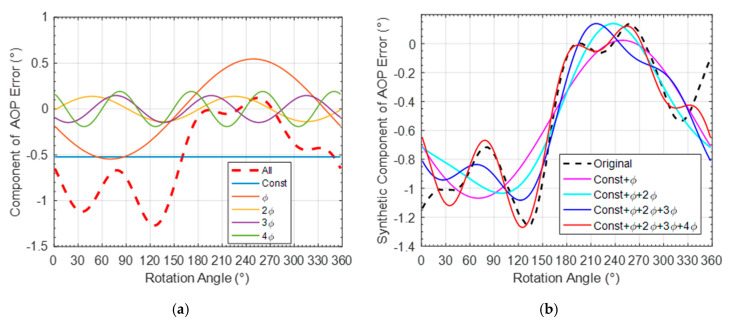
Spectral analysis of the outdoor AOP error. (**a**) spectrum component; (**b**) synthetic component of AOP error.

In addition, it can be seen that the calibrated polarization accuracies achieved in outdoor environment are worse than that in indoor environment. To analyze the influence of the complex outdoor interference on the polarization information acquisition, the error of calibrated AOP was analyzed with the frequency spectrum, as shown in Figure 14a (ϕ is the frequency of platform rotation).

Figure 14b is the synthetic component of the AOP error with different added harmonic components. It can be seen that the AOP error after calibration has a constant error of about 0.5°. and in the first four harmonic components, ϕ frequency harmonic component has a maximum amplitude. In the future work, the influence of platform rotation and outdoor environment factors on the sensor will be further analyzed to facilitate the practical navigation application of the bionic polarization sensor.

## 5. Conclusions

In this study, a new calibration method for camera-based bionic polarization sensor was proposed to account for the inconsistent extinction ratio of polarizer. According to the characteristic of the sensor, an extinction ratio coefficient was introduced to the calibration model. To achieve high accuracies of the estimated model parameters, an iterative least square calibration algorithm considering both AOP and DOP errors was proposed. Both indoor and outdoor calibration experiments were carried out to evaluate the proposed calibration model and calibration method. The results showed that 0.04° (1 σ) calculated AOP accuracy with 0.10° maximum absolute error can be achieved after the indoor calibration and 0.71° (1 σ) AOP accuracy after the outdoor calibration. To further validate the efficiency of the proposed calibration method, three different comparative experiments were conducted. It showed that the calibration model with extinction ratio coefficient can effectively improve the accuracies of the calculated AOP and DOP. The standard deviations of DOP and AOP were reduced by 46.5% and 2.0%, respectively.

## Figures and Tables

**Table 1:** Statistics comparison of AOP error under different light-intensity levels.

Intensity Level (lux)	140	300	500	700
**SD (°)**	0.14	0.10	0.11	0.07
**MAXE (°)**	0.38	0.24	0.26	0.21

**Table 2:** Orientation-angle error comparison.

Calibration	Original	Step 1	Step 2
**SD (°)**	2.62	0.22	0.04
**MAXE (°)**	7.39	0.47	0.10
**MAE (°)**	3.51	0.20	0.03

**Table 3:** Description of the three cases.

Types	Model	Method
**Case1**	without ER	based solely on error of AOP
**Case2**	with ER	based solely on error of AOP
**Case3**	with ER	based on error of both AOP and DOP

**Table 4:** Statistics comparison of DOP error.

Types	Case1	Case2	Case3
**SD (×10^−3^)**	4.02	2.15	1.04
**MAXE (×10^−3^)**	7.70	3.93	2.98
**MAE (×10^−3^)**	3.71	1.84	0.82

**Table 5:** Statistics comparison of AOP error.

Types	Case1	Case2	Case3
**SD (×10^−2^ °)**	4.08	4.00	4.06
**MAXE (×10^−2^ °)**	11.3	10.5	10.3
**MAE (×10^−2^ °)**	3.20	3.19	3.22

**Table 6:** Statistics comparison of AOP error.

Calibration	Original	Step 1	Step 2
**SD (°)**	2.13	1.16	0.71
**MAE (°)**	2.73	1.32	0.68
